# Industrial Fatigue and the Productive Body: the Science of Work in Britain, *c*. 1900–1918

**DOI:** 10.1093/shm/hkx077

**Published:** 2017-09-18

**Authors:** Steffan Blayney

**Affiliations:** Flat 31 Gilesmead, 79 Camberwell Church Street, London, UK

**Keywords:** industrial fatigue, industrial physiology, productive body, François Guéry, Didier Deleule

## Abstract

This article examines the emergence of ‘industrial fatigue’ as an object of medico-scientific enquiry and social anxiety in early-twentieth-century Britain. Between 1900 and 1918, industrial fatigue research became the basis of a new science of work, which I term ‘industrial physiology’. Drawing on François Guéry and Didier Deleule, I argue that industrial physiology is best understood as a science of ‘the productive body’. The worker was an object for medico-scientific intervention only insofar as they represented a constituent part of the machinery of industrial labour, while the individual body was, in turn, reimagined as a productive system in microcosm. In this context, industrial fatigue—defined as diminished capacity for productive work—emerged as the emblematic pathology of industrial civilisation. By 1918, it had become the central category in the scientific articulation of a conception of the body in which health was equated squarely with productive capacity.

‘So tired!’ is the cry of thousands of men, women and young persons at the close of the working day. How to meet the complaint and to remove its cause are among the problems of the present age. It would seem as if the stress of modern times was becoming too great, and as if the strain of industrial methods through improved machinery was becoming more than human strength can bear. (Thomas Oliver, *Journal of State Medicine*, 1914)

With this warning, the distinguished physician and professor of physiology, Sir Thomas Oliver, began a 1914 article entitled ‘Occupational Fatigue’.^1^ While in part echoing Victorian anxieties linking the pressures of modernity with bodily, social and cultural exhaustion, the exclusive focus of Oliver’s article on the factory and on the industrial working class represents an important shift in scientific and political discussions of fatigue at the turn of the twentieth century.^2^ While the concept of fatigue—understood as the exhaustion of the body’s capabilities for effort and exertion—entered British scientific and medical discourse in the second half of the nineteenth century, the specific construction of Oliver’s title—or its far more common variant, *industrial* fatigue—cannot be found prior to the twentieth.

In the early years of the twentieth century the category of industrial fatigue became the basis of an eclectic but coherent field of medico-scientific enquiry, which I will refer to as ‘industrial physiology’. It took shape in a handful of scientific texts, research committees, and government-sponsored investigations in the years before the First World War and played an important role in debates about hours and conditions of work across a number of industries. During the war, it was catapulted to prominence with the formation of the Health of Munition Workers Committee appointed by the government to ‘consider and advise on questions of industrial fatigue’. By 1918, with the conversion of this wartime body into a permanent Industrial Fatigue Research Board, it had become firmly established within scientific and political discourse.

While the term ‘industrial physiology’ was not widely used in Britain in the period, I have taken my lead from the contemporary American physiologist Frederic S. Lee who, in an article of 1919, suggested its use to denote a new scientific approach to work, which he saw as having risen to prominence during the First World War. For Lee, the British pioneers of work science were exemplary figures in this new approach, and his broad definition corresponds to the way in which I wish to use the term. ‘I have called this new science “industrial physiology”,’ he wrote, ‘because this term seems to me to be the most appropriate single term to use in discussing this new phase of the application of scientific method to the solution of human problems. By it I mean to designate the sum of knowledge pertaining to the working of the human mechanism in industrial activity.’^3^

While by no means a formal grouping, the individuals who formed the constituency of industrial physiology nonetheless developed a shared conceptual apparatus which justifies their being discussed as a coherent group, with many, in addition, sharing institutional affiliations. While not all were academic physiologists (industrial physiology’s spokespeople also included physicians, economists, social reformers, politicians, government officials, industrialists, and trade unionists), all were united by the conviction that industrial work should be understood primarily in physiological terms. They sought to apply the science of the human body to the problems of industrial civilisation. Through research into the physiological laws governing industrial work and fatigue, they argued, labour could be organised so as to maximise the productivity of the worker and the prosperity of the nation.

As Richard Gillespie has argued, industrial fatigue was a central locus for negotiations of expertise and professional authority in the early twentieth century. For laboratory scientists, it represented a chance to ‘define a new social role for physiology’, to expand disciplinary boundaries and claim legitimacy to comment on social and political issues.^4^ In turn, through the adoption of a physiological vocabulary, social reformers could claim authority for their various proposals in an era in which policy was increasingly being dictated by scientific expertise. Likewise, for employers and the state, the discourse of industrial physiology could be used to justify the implementation of means by which to increase profits and productivity, all the while appealing to the objective authority of scientific knowledge.

The scientific approach to the labour process which characterised industrial physiology in Britain had much in common with that of the contemporary ‘science of work’ emerging in Continental Europe, the history of which has been detailed by Anson Rabinbach.^5^ In the late nineteenth and early twentieth centuries, Rabinbach shows (focusing chiefly on French and German developments), human labour was made an object of scientific study. New theories of human energy turned fatigue—understood as the body’s declining capacity to convert energy into useful work—into a major medical and social problem. Scientists and social reformers alike were motivated by the utopian goal of the ‘body without fatigue’ and with it the elimination of the material and symbolic limits on efficiency, productivity and social progress.^6^

This European science of work was undoubtedly influential in the development of industrial physiology in Britain, with scientists and reformers reading, and often citing, works by continental authorities on fatigue, in particular the Italian physiologist Angelo Mosso, whose 1891 monograph *La Fatica* was translated into English in 1904.^7^ At the same time, however, the British science of industrial fatigue was significantly shaped by domestic political culture. In particular it can be seen as influenced by the ideology of ‘national efficiency’, which Geoffrey Searle has identified as rising to prominence in the period from 1899 to 1914. If ‘efficiency’, as Searle has argued, was the ‘political catchcry’ of early twentieth-century Britain, then industrial fatigue was its dark and disturbing underside.^8^ Behind optimistic visions of a scientifically ordered society, economic progress, and national prosperity, lurked the shadow of an industrial workforce dogged by chronic exhaustion and unable to sustain its capacity for work.

Like other discourses associated with the national efficiency movement, such as eugenics and social hygiene, industrial physiology was predicated on a powerful homology between the biological and the social. Indeed, the construction ‘industrial fatigue’—affixing a socioeconomic descriptor to a medical category—collapsed the two in a single stroke. National efficiency, argued the industrial physiologists, began with the body of the worker. By eliminating fatigue at a biological level, the social, political and economic energies of the nation as a whole could be channelled to their maximum efficacy. As such, its proponents claimed authority not so much as guardians of the flesh-and-blood body of the individual worker, but as technicians of what François Guéry and Didier Deleule have called ‘the productive body’.^9^

First published in French as *Le corps productif* in 1972, Guéry and Deleule’s *Productive Body* only appeared in a complete English translation for the first time in 2014. Building on Karl Marx’s analysis of the labour process in *Capital*, Guéry and Deleule seek to understand how the body is produced and understood in capitalist societies.^10^ Central to their argument is a tripartite classification of three overlapping categories of ‘body’: the ‘biological body’, the ‘social body’ and the ‘productive body’. The biological body, in Guéry and Deleule’s schema, refers simply to the ‘human apparatus’ prior to its investment with social meaning.^11^ The ‘social body’ then describes the ‘interweaving of society and the body’. As in the phrase ‘the body politic’, the term can be seen as referring both to a mass of bodies linked together by a social bond, and to a representation of the individual body as it is understood within a particular society.^12^ Finally, the ‘productive body’ is the socialisation of the body which is unique to capitalism: a social body organised for capitalist production. The term refers at once to the collective mass of the labour force as a whole (analogous to Marx’s notion of the ‘collective labourer’), and, at the same time, to a particular ideological representation of the individual body under capitalism.^13^

For Guéry and Deleule, following Marx, ‘productivity’ refers not simply to the capacity for creative labour, but specifically to the ability to produce surplus-value for a capitalist employer—that is, to a particular condition of alienated labour.^14^ Under capitalism, Guéry and Deleule argue, ‘there is a tendency … toward the conversion of human material into productive-form’. Individual bodies are reimagined in terms of their economic potentials, and integrated ‘within the productive body as elements of production’.^15^ The result is ‘a representation of living beings in which work’s production is constitutive of the perceived being’, such that the human body appears as a ‘pure work machine’.^16^

If physiology is the scientific study of the human body, then *industrial* physiology, as it emerged in Britain in the early twentieth century, was a science of the *productive* body. While its vocabulary was biological, it conceived of the human body wholly in terms of its economic attributes. The worker’s physical capabilities were reduced to productive capacities. The worker was an object for medical and scientific intervention only insofar as he or she represented a constituent part of the machinery of industrial labour, while the individual body was, in turn, reimagined as a productive system in microcosm.^17^ In this context, industrial fatigue emerged as the emblematic pathology of modern industrial civilisation: a problem not only, or even primarily, of biology, but of productivity.

This article traces the emergence of the discourse of industrial physiology through the development of its central category: industrial fatigue. In the early twentieth century, I argue, this category became the basis of a new British science of work and of the working-class body. The emblematic pathology of the productive body, industrial fatigue threatened to put at risk economic growth, national prosperity and social stability. Anxieties about the exhaustion of the working population were mobilised to legitimate an extension of physiological knowledge from the laboratory into society, and to justify interventions into the organisation of the workplace by a new breed of industrial experts, placing scientific knowledge at the centre of debates about management and the organisation of work. By the end of the First World War, I argue, the opposition between efficiency and fatigue articulated by industrial physiologists had become the basis of new scientific conception of the body in which health was equated squarely with productive capacity.

## The Emergence of a Scientific Object

To the extent that historians have taken account of the rise of industrial physiology, it has usually been seen as a well-intentioned—if perhaps long overdue, or insufficient—attempt to address a self-evident and pre-existing problem. In this context the development of a science of work has been viewed as merely the accumulation of empirical knowledge, and its application to the problem of ‘industrial fatigue’.^18^ As Bruno Latour and others have emphasised, however, a ‘scientific object’ (such as industrial fatigue) cannot be simply ‘extended into the past’ at no cost to our historical analysis.^19^ Objects of scientific study should be seen, not as a pre-existing entities, lying in wait for scientists to discover them, but as historical formations, fundamentally tied to the contexts in which knowledge about them is produced and put to use. From this perspective, to state—as Arthur McIvor does—that ‘Industrial fatigue … was widely prevalent in nineteenth-century industry’ is therefore potentially misleading.^20^ It is not simply that the term ‘industrial fatigue’ was not used before the twentieth century, but that its introduction represented the arrival of a new way of conceptualising the working body that was previously unavailable.

Whether or not workers in nineteenth-century factories suffered the effects of long hours and poor conditions, it was not until the twentieth century that the concept of ‘industrial fatigue’ was deployed as a means to describe this state of affairs, or to justify interventions into the workplace. Nineteenth-century reformers who sought to address questions of long hours and overwork conceived of the problem in different terms. In 1893, for example, a reduced, forty-eight hour working week was trialled by William Mather at the Salford Iron Works, demonstrating that a cut in hours did not lead to a corresponding fall in production.^21^ While his conclusions were often cited by proponents of industrial physiology in the twentieth century, Mather did not justify his own experiments in the scientific vocabulary that would come to dominate debates about overwork after 1900. In his published account of the experiment Mather stressed ‘the moral, as much as the physical, effect’ of shorter hours.^22^ He made no attempt to ground his results in physiology and did not draw on any scientific sources to back up his conclusions. The words ‘fatigue’ and ‘efficiency’ do not appear anywhere in the text. For other ‘enlightened employers’ of the late-nineteenth and early-twentieth centuries who advocated shorter hours, the rhetoric was also philanthropic rather than physiological. Edward Cadbury, for example, despite later adopting the language of physiological efficiency, was, as late as 1912, still advocating shorter hours for young workers, combined with the provision of educational facilities, on the basis ‘that they may have as varied and full a social life as possible’.^23^

The political context of the early twentieth century provided the conditions for concerns about overwork to be translated into the language of industrial physiology. The period from the beginning of the twentieth century to the start of the First World War saw the development of new discourses about the working-class body, with Social-Darwinist inspired movements of eugenics, ‘social hygiene’ and ‘national efficiency’ influencing public debate across the political spectrum.^24^ Revelations about the poor physical condition of army recruits during the Boer War (1899–1902), provoked widespread anxieties about the ‘physical deterioration’ of the urban working class. Increasingly, British national and imperial greatness, as well as economic prosperity and productivity, was seen to turn upon the physique of its citizens. As one author put it in 1907:

The health of the people of a country stands foremost in the rank of national considerations. Upon their health depends their physical strength and energy, upon it their mental vigour, their individual happiness, and, in a great degree, their moral character. Upon it, moreover, depends the productivity of their labour, and the material prosperity and commercial success of their country. Ultimately, upon it depends the very existence of the nation and of the Empire.^25^

The development of industrial physiology—collapsing as it did, the distance between the biological body and the productive body—was shaped by such concerns. If governments were sincere in their concerns for the ‘physical condition of our people’ argued the educationist Margaret Drummond in 1905, then there was a ‘pressing need for a science of fatigue’.^26^

The scientific elaboration of the concept of industrial fatigue was, from the start, inseparable from calls for its practical application as a tool of industry and of the state. Indeed, the first use of the phrase in a British context occurred in the context of a call for state intervention. At the Thirteenth International Congress of Hygiene and Demography, held in Brussels in September 1903, the section of ‘Industrial and Professional Hygiene’ resolved that governments should facilitate research on ‘the problem of overwork as a result of industrial labour’.^27^ Reporting back to Parliament, British delegates Adelaide Anderson and Thomas Legge (both Home Office factory inspectors), testified that the Congress had called for ‘investigations on the subject of industrial fatigue’.^28^

Giving the proposals their full approval, Anderson and Legge stressed the potential practical utility of fatigue research for the state. ‘[C]learly,’ they argued, ‘were it possible to estimate with some degree of precision the amount of fatigue employed in manufacturing processes, positive data on which to base legislation would be available.’^29^ Anderson expressed similar sentiments in her evidence to the Inter-departmental Committee on Physical Deterioration, and in its report of 1904, the Committee deemed it ‘highly desirable that there should be a strictly scientific enquiry into the physiological condition and effects of over-fatigue, as recommended by the Brussels Congress’.^30^

## Institutionalising Fatigue Research

Despite such hopes, it would be almost a decade before a government-led study of industrial fatigue was put in place. In the meantime, however, the concept of industrial fatigue was taking shape, both in Britain and internationally. The topic of fatigue was again discussed at the International Congresses of 1907 and 1912, in Berlin and Washington, DC, respectively. Among those delivering papers at the latter event was the American campaigner for labour reform Josephine Goldmark, whose *Fatigue and Efficiency* was published in the same year. Goldmark’s book, which aimed to ‘explain the phenomena of overwork in working people’, and thus provide ‘a scientific basis of legislation’, was widely read in Britain and influenced a number of reformers.^31^ In the *Sociological Review*, the social investigator, feminist and socialist, Bessie Hutchins, praised it as ‘the first systematic treatise on the dynamic relation of the worker to the work’ and welcomed Goldmark’s proposals for a harmonious—and productive—marriage of scientific expertise and factory legislation.^32^ Whereas previously reformers could rely only on the ‘mercy’ or ‘pure philanthropy’ of lawmakers and employers, Hutchins reasoned, a scientific investigation of work would place at their disposal ‘detailed facts and figures’ and ‘a considerable body of scientific observation’ to show that a reduction in the hours of work would be beneficial not only to workers, but to employers’ profit margins. With the development of a science of fatigue, Hutchins posited, previously intractable problems of industrial relations could be settled by an appeal to pure science. ‘The relation of output to effort is fast coming well within the range of scientific measurement,’ she declared, optimistically predicting that ‘eventually a scientific formula will be found for the relation of productivity to effort, and that it will involve something like another industrial revolution.’^33^

The development of a scientific formula for the relationship between work and fatigue, and of a reliable means by which the latter could be quantified and measured, were the key desiderata of early research into industrial fatigue, and were the goals of the first major foray into fatigue research by the British state. In 1912, Albert Stanley Kent, professor of physiology at the University of Bristol, was appointed by the Home Office to conduct an ‘investigation of industrial fatigue by physiological methods’. His brief was ‘to discover a test for recognising the presence, and a gauge for estimating the degree, of fatigue as met with under factory conditions’, which could then be applied by the Factory Inspectorate as a means by which to judge the effects of long hours and overwork.^34^ The science of industrial fatigue, as Kent explained in his first report, was largely a practical, rather than theoretical, endeavour. From ‘the industrial point of view’, it was less important to understand the physiological nature of fatigue than to determine the extent to which productivity was affected by overwork.^35^ Through careful surveillance and control of the factory environment and hours of work, the conditions for ‘the attainment of maximum output’ could be scientifically arrived at.^36^

The increasingly practically-minded nature of fatigue research in the first decades of the twentieth century is neatly captured in a comparison of two research committees on the subject, formed within a few years of each other by the British Association for the Advancement of Science. The first, established in 1908 and chaired by Charles Sherrington, was organised under the Association’s physiology section and preoccupied itself largely with laboratory experiments into muscular and mental performance.^37^ The second, established in 1913, was established not under the physiology section, but that of ‘economic science and statistics’. Chaired by the philosopher and professor of political economy John Henry Muirhead, it took as its object of investigation ‘fatigue from the economic standpoint’.^38^ In actuality, the second committee was eclectic in composition, comprising not only economists and statisticians, but also physiologists, psychologists, physicians, and other miscellaneous experts and enthusiasts in the burgeoning field of fatigue and efficiency.^39^ The factory inspector and veteran of the Brussels Congress Adelaide Anderson was a member, as was the ‘progressive’ industrialist Edward Cadbury, while its energetic organising secretary was the same Bessie Hutchins who had enthused over Goldmark’s vision of a scientifically-ordered and maximally efficient factory in the *Sociological Review*. Kent too collaborated with the Association committee in tandem with his physiological investigations for the Home Office.

In truth, the science represented by the British Association Committee on Fatigue from the Economic Standpoint—as well as by Kent’s *Investigation of Industrial Fatigue by Physiological Methods*—was neither physiology nor economics, but a novel combination of the two: the emerging hybrid discipline I have referred to as industrial physiology. In terms of its scientific foundations, industrial physiology did not significantly depart from the explanatory frameworks and vocabulary of late-nineteenth-century physiology. However, it can be differentiated from earlier writings on fatigue both by its exclusive focus on industrial work and the working class, and by a broad shift in emphasis from the theoretical to the practical; from the internal, biochemical workings of fatigue to its external, economic effects.

Above all, the industrial physiology articulated in the years before the First World War was characterised by a conviction that fatigue was an entirely *objective* phenomenon, subject to precise measurement and quantification. While the article on ‘occupational fatigue’ which opened this article, began with the anguished cry of the exhausted worker—‘So tired!’—such a focus on subjective suffering was in fact, by 1914, extremely uncommon. From the standpoint of industrial physiology, the sensations of the worker were irrelevant to the scientific study of fatigue. One worker might complain of fatigue while displaying no measurable decline in work performance, experiments recorded, whereas another’s work may drop off without their noticing any sensations of tiredness. For all practical purposes , as the reports of both investigations made clear, fatigue was to be understood as ‘a lessened capacity for work’, and measured in terms of declining ‘output’.^40^

## War and Industrial Fatigue

In Kent’s investigations for the Home Office, and in the work of the British Association’s Committee on Fatigue from the Economic Standpoint, the concept of industrial fatigue which had been developing from the start of the twentieth century was beginning to solidify. In these institutional settings, the hybrid discipline of industrial physiology was taking shape. Broad agreement was reached about how industrial fatigue was to be defined, and how the work of industrial physiology was to be carried out. Specifically, industrial fatigue was an objective physiological condition caused by industrial work and affecting the working-class body. It was defined as the diminished capacity for work, and, through analysis of empirical or statistical data relating to factory work, it could be measured in terms of declining work performance.

By 1914, however, industrial fatigue was still a relatively minor issue. Its emergence as an object of scientific enquiry and the proliferation of experimental research had not been matched by a similar level of political attention. While government departments and a few employers were, in the first two decades of the twentieth century, beginning to show an interest in the optimisation of the working body, discussions of fatigue were still largely limited to the small circle of physiologists, economists and factory reformers who comprised the core constituency of industrial physiology. In addition, as Arthur McIvor has shown, the impact of shorter-hours experiments and scientific fatigue research on the organisation of British industry before 1914 was minimal.^41^ By the time the first interim reports of Kent and the British Association committee were published, however, international events had made industrial fatigue an urgent matter of public debate. Britain’s entry into the First World War, and the demands it placed on domestic industry, made the question of the limits to the body’s productivity a question of national political—perhaps even existential—significance, and provided the conditions for the first large-scale intervention on the part of the state.

The motivation to action was a crisis, not of workers’ welfare, but of production. The ‘shell crisis’ of May 1915, in which the lack of artillery shells being provided to the front line was exposed, caused a national scandal, and played a large role in the fall of the Liberal government.^42^ One of the first items of business for the new Coalition Government, formed by Prime Minister Herbert Henry Asquith in the same month, was the creation of a new Ministry of Munitions, specifically to manage the production and distribution of munitions for the war effort. David Lloyd George, who had made the shell crisis his personal cause, resigned his post as Chancellor of the Exchequer to head the new department.^43^ The express purpose of the Ministry was to increase production. It organised the building of new government factories and the conversion of existing engineering workshops for the production of armaments. The Munitions of War Act of July 1915 empowered the Minister to declare any private munitions factory a ‘controlled establishment’, bringing it under the direct control of the Ministry, with powers to control profits and wages, and requiring employers to provide detailed information about numbers of workers employed, the conditions of work, and the hours of labour.

In addition to the expansion of production and the mobilisation of new labour, the Ministry was also concerned with increasing the productivity of those workers already employed in its factories, and the emergency powers granted to it by the Munitions of War Act—as well as the extreme conditions of overcrowding, poor sanitation and long hours brought on by the pressure of munitions work in the early years of the war—provided an unprecedented opportunity for the state to implement a large-scale investigation into the efficiency of the working body. In September 1915, Lloyd George appointed the Health of Munition Workers Committee (HMWC), to ‘consider and advise on questions of industrial fatigue, hours of labour, and other matters affecting the personal health and physical efficiency of workers in munitions factories and workshops’.^44^

Like the pre-war British Association committee on fatigue, the HMWC contained an eclectic mix of scientific, medical, industrial and administrative expertise.^45^ On the medico-scientific side, the physiologists Walter Morley Fletcher and Leonard Hill (both representatives of the recently-formed Medical Research Committee), were joined by Arthur Boycott, a professor of pathology at the University of Manchester, and by Thomas Barlow, formerly the personal physician of Queen Victoria. The interests of employers were represented by Samuel Osborn, of the engineering firm Samuel Osborn & Co., and those of workers, nominally, by the trade unionist, Labour MP and efficiency enthusiast, John Robert Clynes. The Home Office Factory Department supplied Medical Inspector Edgar Collis and Senior Lady Inspector Rose Squire, while further female representation was provided by the former factory inspector, May Tennant. The position of chairman was taken by Sir George Newman, medical officer to the Board of Education, and Edward Pelham, also from the medical department of the Board of Education, was recruited as secretary.^46^

As well as this permanent core, the HMWC also employed the services of a number of other experts to conduct a series of investigations into fatigue, hours of work and factory conditions in government-controlled factories. The eclectic range of investigators included the economist Philip Sargant Florence, who had conducted the bulk of the research for the British Association committee on fatigue, the medical statistician Major Greenwood, and the philosopher Thomas Loveday. The most energetic researcher, personally authoring three of the HMWC’s memoranda, was the Oxford physiologist Horace Vernon, who offered his services to the Committee after volunteering at a Birmingham munitions factory during the university vacation, experiencing first-hand the effects of a 74½-hour nominal week, plus overtime.^47^ The results of the HMWC’s research formed the basis of 21 memoranda on a variety of subjects produced between 1915 and 1918 (all but one of which were published), as well as two larger published reports, and a specially prepared handbook for factory managers.

Historians have rightly stressed the importance of the First World War in bringing the question of industrial fatigue to national attention in Britain.^48^ As one commentator remarked in 1917, ‘the war has caused us to give more attention to fatigue during the past two years than it has received from us during the preceding half century’.^49^ In the work of the HMWC the discourse of industrial physiology which had been developing in Britain over the previous decade or so would receive its fullest, and most influential, articulation. In theoretical terms—that is, in the scientific definitions and explanations of fatigue they advanced—the HMWC made few innovations. However, the problems established by industrial physiology were given new salience in a transformed political context. If early-twentieth-century concerns about the physical deterioration of working-class bodies, encapsulated in the new concept of industrial fatigue, had collapsed the physiological with the social, the effect of the war was to invest the metonymic relation between biological body and productive body with a new patriotic significance. As Britain’s collective industrial power was mobilised for an imperial war, the body of the factory worker—and the munition worker in particular—became a physical embodiment of national strength. The productive body became a military-industrial complex. Fatigue now became an ‘urgent national problem’, potentially representing not simply the decline of profits, but the difference between winning and losing the war.^50^ ‘The health of the munition worker,’ as chairman George Newman put it, was ‘just as important to the Nation as the health of the soldier.’^51^ The elimination of fatigue was central to ‘the vigour, strength and vitality of the nation’.^52^

Just as early industrial physiology had predicated its social utility on providing scientific answers to practical questions of industrial organisation, the HMWC conceived of their work as an applied science: ‘a cross breed’, as Newman described it, ‘between research and administration’.^53^ While the members of the HMWC—along with the ministry’s Welfare Department, established in December 1915 on the basis of an HMWC investigation into welfare supervision, and headed by the Quaker industrialist Seebohm Rowntree—often couched their work in terms of worker’s welfare, their primary purpose was unambiguously the maximisation of productivity in service of the war effort.^54^ The HMWC, as the Ministry of Munitions’ Christopher Addison reminded one committee member in September 1915, was appointed by the ministry ‘with a view to securing an improved output of munitions of war’.^55^

The discourse of industrial physiology—defining fatigue precisely as an objective decline in output—fitted such imperatives perfectly. In a memorandum on ‘Industrial Fatigue and its Causes’, the HMWC defined fatigue as ‘the sum of the results of activity which show themselves in a diminished capacity for doing work’.^56^ Subjective manifestations of fatigue were irrelevant to its objective course, and were not worthy of scientific consideration. The memo continued:

In ordinary language fatigue is generally associated with familiar bodily sensations, and these sensations are often taken to be its measure. It is of vital importance for the proper study of industrial fatigue, however, to recognise not only that bodily sensations are a fallacious guide to the true state of fatigue which may be present, and a wholly inadequate measure of it, but also that fatigue in its true meaning advances progressively, and must be measurable at any stage by a diminished capacity for work, before its signs appear plainly, or at all, in sensation.^57^

While the committee’s chief researcher Horace Vernon may have been able to draw on his own experience of subjective fatigue as a volunteer munitions worker, he was careful not to let such considerations influence his work for the HMWC. If working capacity was not diminished, as he later clarified, then any apparent fatigue, even though it might produce ‘severe subjective sensations in the worker’ could be described as neither ‘abnormal’ nor ‘pathological’, and ‘serious objection could not be taken to it on the ground of the sensations produced’.^58^ Fatigue as pathology, in other words, was not an internal affliction of the individual, but an external condition of the productive body, measurable objectively in terms of declining work rate. ‘In practical usage,’ as Alan Derickson has argued, ‘it was only a short step to defining fatigue as diminished output’, with the crucial implication that ‘if tired employees could be driven by threats, stimulants, financial incentives, nationalistic appeals, or machine pacing to maintain output throughout their work shifts, no fatigue existed’.^59^ As a later memo on hours of work confirmed, if efficiency could be maintained, the HMWC would ‘raise no *a priori* objections to any given number of hours, however long’.^60^

‘The true sign of fatigue is diminished capacity,’ explained the HMWC’s memo on industrial fatigue, ‘and it follows from what has been said that measurement of output in work will give the most direct test of fatigue.’^61^ It is worth emphasising the circular, reifying logic of such claims. Industrial fatigue was proposed as an explanation for declining productivity, then measured in terms of the very decline it was supposed to explain. As a result, fatigue, rather than an internal physiological phenomenon, was now in practice completely externalised, defined completely in economic terms, quantifiable in the number of units a worker could produce in a given period of time.

As well as the so-called ‘direct’ measure of output, the memorandum on fatigue detailed a number of ‘secondary’ methods by which a measurement of fatigue could be obtained—foremost among them the incidence of ‘accidents’ and of ‘spoiled work’—again corresponding to productivity rather than biology. Ideally, the Committee explained, measurements should be taken without the workers being aware of it, so as minimise any subjective influence on the results. This was a physiology, then, which had no need of bodies. Direct experimental research on workers (in the form, for example, of tests of muscular strength or reaction time) was rare. Instead, as numerous reports and research manuals set out, it was preferable to rely on statistical data collected from records kept by factory management.^62^

Rather than focusing on the biological body (which was always kept at a distance), industrial physiology sought to isolate and measure the *productive* body in its pure form—as a statistical composite and mathematical average—at once derived from, yet radically divorced from, the flesh-and-blood bodies of the workers themselves. For industrial physiology, the most direct way to observe the working body was not to observe it all, relying almost exclusively instead on abstract representations of pure productivity. The subjective sensations of the body entered into the work of the HMWC only as a disruptive influence, the effects of which needed to be minimised in the practice of proper scientific methodology. Indeed, the purpose of the scientific method was to render such subjective feelings invisible.

## Physiological Management

When it came to the practical recommendations of the HMWC, such a conceptual framework had significant implications. If workers were unable to accurately recognise their own fatigue then their opinions as to the effects of work on their own bodies could have no legitimate bearing on industrial questions, such as factory conditions or hours of work. As the memorandum on fatigue explained:

[D]uring the continued performance of work the objective results of nervous fatigue precede in their onset the subjective symptoms of fatigue. Without obvious sign and without his knowing it himself, a man’s capacity for work may diminish owing to his unrecognised fatigue.

After a certain point, the memo argued, the workers’ time ‘begins to be uneconomically spent’. It was the responsibility of ‘scientific management’—and not the worker—to determine this point and to ‘determine further the arrangement of periods of rest in relation to spells of work that will give the best development … of the worker’s capacity’.^63^ ‘If the operatives are left to themselves,’ recorded another memorandum, on the subject of hours of work, ‘they take rests at irregular and often unsuitable times. Hence it would be much better if the rest pauses were chosen for them.’^64^ The organisation of work was a purely technical question. Questions of hours of work, the intensity of labour and distribution of rest spells, were not to be negotiated between management and labour, but determined by objective scientific knowledge and the expertise of industrial physiology.

In making such recommendations, the HMWC were keen to distance themselves from the contemporary systems of workshop rationalisation that went by the name ‘scientific management’. While, as the above-quoted passage shows, they were not always shy of using the term, the proponents of industrial physiology were always careful to distinguish their own work from the ‘American’ forms of scientific management associated with Frederick Winslow Taylor and his followers.^65^ In part, this was down to an awareness of the widespread suspicion among workers and unions, who broadly viewed scientific management and its methods (particularly time and motion study) as little more than a means for employers to extort a greater intensity of work from employees. However, the rejection of ‘American’ scientific management also provided an important point of contradistinction by which industrial physiology could define its own particular expertise.

The British advocates of industrial physiology characterised the American-inspired ‘efficiency engineers’ as presenting a simplistic, reductive, mechanical view of the worker, offering crude one-size-fits-all solutions which were concerned more with increasing profits in the short term, through ‘driving’ labour to breaking point, than with determining the true scientific principles and physiological laws which would ensure the maximum of efficiency. In short, as the British Association Committee on Fatigue from the Economic Standpoint concluded, in failing to recognise the true physiological basis of the labour process, the problem was that ‘scientific management’ was simply not scientific enough: ‘Scientific Management has perhaps not spent enough time searching scientifically for the laws of fatigue before setting its standard intensity of work,’ the Committee protested, ‘yet, if once these laws are discovered, then it is only to *a really scientific management* that we can look for the application of the discovery’.^66^ While acknowledging research by Taylor and others on the relation of the distribution of breaks in work to output, the HMWC’s final report likewise concluded that this was ‘another problem which has never yet been *scientifically* explored’.^67^

For the proponents of industrial physiology, it was not that the working body could not be thought of in mechanical terms, but only that previous researchers had failed to appreciate the complexity of the machine they were dealing with; the sheer number of variables which affected the performance of its work. ‘[T]he human machine,’ wrote Kent in 1917, ‘infinitely more complex and highly tuned than any work of man, is correspondingly delicate and dependent for its efficiency upon suitable surroundings.’^68^ In principle, the working body obeyed strictly predictable physical and physiological laws, yet these were often hard to determine in practice due to complex and interconnected influences of psychology and environment. In the interwar period, consideration of these complicating factors would lead to a greater focus on the worker’s psychology and the ‘human factor in industry’.^69^ In the HMWC, tensions between the view of the worker as a complex human being and a simple instrument of production led to sometimes paradoxical statements, such as chairman George Newman’s caution that ‘the worker is not a machine, and cannot be so treated without grave loss of efficiency’.^70^

Despite dressing criticisms of scientific management with references to workers’ well-being, industrial physiology made it explicit that the problem of scientific management wasn’t that it increased output to the detriment of worker’s physical and mental health, but that, in the long run, it *failed* to increase output. And, further, that only a properly scientific application of the principles of industrial physiology could secure a permanent increase in productivity. While the object of American so-called scientific management was simply a direct increase of output in the short term, explained the HMWC’s Walter Fletcher in an early memorandum to the Ministry of Munitions, ‘The object of scientific *physiological* management is to secure the optimum physiological efficiency and the maximum output’ over long periods of time.^71^ The characteristic innovation of industrial physiology—which reached its apogee in Britain with the work of the HMWC—was to insist that the two were in fact identical: that the physiological optimisation of the worker’s body was one and the same with the maximisation of his or her productivity.

This conceptual elision had important political consequences. If the protection of workers’ bodies, the reduction of their fatigue and the enhancement of their productivity were one and the same, then disputes between capital and labour over working conditions or the length of the working day were illusory, and could be satisfactorily resolved in the best interests of all parties by the mediation of impartial scientific expertise. ‘The problem of scientific industrial management,’ as the HMWC repeatedly asserted, ‘dealing as it must with the human machine, is fundamentally a problem in industrial fatigue.’^72^ Conflicts between workers and employers had arisen in the past due to work being organised in contravention of ‘physiological law’, and industrial physiology promised ‘a hearty co-operation between employers and employed, in the task of finding the optimum conditions of work for the benefit of both’.^73^ As Kent reflected in 1920:

Yet it may be said with certainty that the best result, in the sense of greatest output with least fatigue, can only be obtained by a careful adjustment of hours of work to the conditions of the operation concerned, and that the real interests of capital and labour, which indeed, in this respect are almost identical, should be secured through such an arrangement based on scientific principles.^74^

Even John Clynes—a socialist and trade unionist as well as an HMWC member—was personally committed to the development of scientifically-managed labour process, in which expertise would replace class struggle as the basis of historical progress.^75^

## Health and Efficiency

‘Sickness’, David Harvey has argued, ‘is defined under capitalism broadly as inability to work.’^76^ Nowhere was this more explicit than in industrial physiology, and the related fields which developed out of it in the twentieth century: industrial psychology, industrial medicine, industrial health. As Steve Sturdy has argued, concerns about the state provision of health and welfare in the first half of the twentieth century frequently equated health with productivity. Likewise, John Pickstone identifies a ‘productionist’ ideology behind twentieth-century British medicine, concerned primarily ‘with the health and strength of workforces and armed forces and with their reproduction’.^77^

In her 2011 monograph *The Rise and Fall of the Healthy Factory*, Vicky Long credits the HMWC with promoting ‘a broadly conceived model of health which embraced physical and mental well-being in all spheres of life’, both inside and out of work.^78^ Such a model, however, is by no means incompatible with an analysis of industrial medicine as a science of the productive body. While the early twentieth century saw increased attention being paid to the health of the worker beyond a narrow focus on industrial disease and compensation, more often than not, as Long makes clear, these broader concerns were underpinned by an ‘economic rationale’ which stressed that a healthy population made for a productive workforce.^79^

When industrial physiology referred to the health of the worker—as in the title of the handbook prepared by the HMWC for munition factory owners, *The Health of the Munition Worker*—it was usually explicit that this meant the health of the worker only insofar as he or she *was* a worker: that is, as far as he or she could meet the minimum bodily requirements to maintain productive efficiency. Industrial health—a term only just coming into use in the early twentieth century—likewise referred to health only insofar as it was relevant to industrial production. Indeed, as in the title of the HMWC’s final report, it usually came as part of a dyad: *Industrial Health and Efficiency*.

For the HMWC investigator Philip Sargant Florence, health could be defined as ‘the actual seat of working capacity’.^80^ ‘Without health,’ a Committee publication began, ‘there is no energy, without energy there is no output.’^81^ In this context, fatigue—defined as ‘diminished capacity for work’—emerged as the all-encompassing pathology of industrial work, effectively describing any impediment which might possibly befall a worker. The opposition between health and infirmity (at least for the working-class body) was resolved into the binary of efficiency and fatigue. While only one of the HMWC’s memoranda was specifically dedicated to the subject of fatigue, it was a constant presence, both symbolically and literally, throughout their reports.^82^ ‘Special industrial diseases’ such as lead or TNT poisoning were an issue only insofar as they might result in ‘interference with output’ (indeed, as Antonia Ineson and Deborah Thom have shown, the HMWC were happy to collude with management to downplay the harmful effects of TNT in order to maintain levels of shell production), while general ‘sickness and injury’ were considered to the extent that they were harmful to ‘industrial efficiency and output’.^83^ Health, like fatigue, could be measured in terms of productivity.

While some historians have emphasised the broad scope of the HMWC’s investigations—taking into account not only the workplace, but a wide range of external considerations such as diet, leisure and home life—it is important to stress the extent to which the unambiguous object of all such investigations was the maximisation of working efficiency.^84^ The conceptual opposition between health (as capacity for work) and fatigue (as its absence) enabled the logic of productivity to take on a comprehensive scope with regard to the body. The body was reimagined as in its very essence productive. ‘The inclination to work rather than to be idle,’ as the HMWC concluded, ‘is a deep-seated natural phenomenon.’^85^ Work became the fundamental *telos* of the body. As such, even those parts of life apparently unconnected with work—indeed, even those ostensibly diametrically opposed to work, such as rest and recreation—could be incorporated within the body’s greater purpose. Inactivity itself was colonised by the logic of productivity, reimagined only as the maintenance and enhancement of the body’s immanent productive capacities. ‘Rest after activity is not a passive state’, stressed the HMWC, ‘but is itself an active process, or a series of active processes, leading to a restoration of the normal capacity for work.’^86^ The worker was not a worker only while ‘at’ work, but was—by virtue of a body whose guiding purpose was to produce output—perpetually caught within a constant ‘rhythm of action and rest’, which could in turn be finely tuned to a state of maximal efficiency.^87^ The study of fatigue and efficiency, argued the HMWC, needed to consider not simply ‘the individual, taken at any one moment’, but ‘his life history, his heredity, his family, his domestic life, his personal habits and customs, his home as well as his workshop’. Not only workers’ bodies, but their whole lives, had to be viewed from the point of view of optimising their productivity, taking into account, as another advocate of ‘industrial medicine’ put it in 1919, ‘every human equation in this problem which affects the health and efficiency of the individual or of the entire group of employees’.^88^

## Conclusion

While acknowledging that in their wartime work they were ‘solely concerned with the factors which are of importance during the present emergency’, and, obviously, only with munitions work, the HMWC and its investigators nonetheless always emphasised that the science of the working body they advanced had a far broader applicability. As the HMWC’s final report, published in 1918 put it, ‘The fact is that this Report of the Committee’s work, though concerned primarily with the munition worker, deals also with vital principles and practical methods affecting all forms of industry.’^89^ By the end of 1917, members of the HMWC were in discussions with government departments about establishing a permanent peace time body through which their research into fatigue and the working body could be continued and expanded beyond munitions factories. In July 1918, this ambition was realised with the appointment of the Industrial Fatigue Research Board. Established under the joint auspices of the Medical Research Committee and the Department of Scientific and Industrial Research, and with the backing of the Home Office, the brief of the new board was ‘to consider and investigate the relations of the hours of labour and of other conditions of employment, including methods of work, to the production of fatigue, having regard both to industrial efficiency and to the preservation of health among the workers’.^90^

Industrial fatigue—an entity which 20 years previously was absent from British scientific or political discourse—was now firmly established within physiological and medical vocabulary, and enshrined in the name of a government institution. While historians have debated the extent to which the HMWC’s recommendations made a significant impact on factory organisation or hours of work at the time, the acceptance of the language of industrial physiology, by the scientific community and by the state, is nonetheless testament to its discursive impact between the start of the twentieth century and the end of the First World War.^91^

The development of industrial physiology was never separate from its practical application, or from the interests of the various groups by whom it was articulated and contested. The science of fatigue and the working body was shaped in response to economic, political, and institutional imperatives. For the discipline of physiology, the problem of industrial fatigue provided a point around which claims of expertise, authority and social utility could be enunciated. By drawing on contemporary rhetorics of eugenics and national efficiency, physiologists presented their discipline as a form of social hygiene, necessary to the continued health of the nation and of the race. For a range of social reformers, the scientific language of physiology and the authority of statistical evidence lent credibility to demands for changes in the organisation of work and working conditions. For industry and government, industrial physiology could legitimate attempts to introduce programmes of scientific management and rationalisation of the body aimed at maximising productivity, all the while claiming to act in the interest of worker’s welfare.

The emergence of the discourse of industrial physiology represented the articulation of a science of what François Guéry and Didier Deleule have termed the productive body. The category of industrial fatigue, collapsing the distance between the biological and the social, placed the body at the centre of debates about work. At the same time, however, it entailed a radically limited understanding of the body as a cog in the industrial machine, far removed from the embodied experience of work. In the final instance, fatigue was not a disease of the worker, but of production. Symptoms were read not from the body, but from its output. For industrial physiology’s proponents, the individual worker was important only insofar as he or she represented a constituent element of the nation’s productive potential, while health itself was reduced to an index of productive capacity.

## Funding

This work was supported by the Wellcome Trust [104927/Z/14/Z].

## Footnotes

1 Thomas Oliver, ‘Occupational Fatigue’, *Journal of State Medicine*, 1914, 22, 347.

2 On Victorian anxieties about fatigue see Anson Rabinbach, ‘The Body without Fatigue: A Nineteenth-Century Utopia’, in Seymour Drescher, David Warren Sabean, and Allan Sharlin, eds, *Political Symbolism in Modern Europe: Essays in Honor of George L. Mosse* (New Brunswick: Transaction Books, 1982), 42–62. On the cultural relationships between fatigue and modernity see Anna Katharina Schaffner, *Exhaustion: A History* (New York: Columbia University Press, 2016).

3 Frederic S. Lee, ‘The New Science of Industrial Physiology’, *Public Health Reports*, 1919, 34, 724. See Alan Derickson, ‘Physiological Science and Scientific Management in the Progressive Era: Frederic S. Lee and the Committee on Industrial Fatigue’, *Business History Review*, 1994, 68, 483–514. In Britain, the term was retrospectively applied to the work of the Health of Munition Workers Committee by the factory surgeon, William Francis Dearden, in an address delivered to the 1924 conference of the Institute of Industrial Welfare Workers. W. F. Dearden, *What Medical Science Can Do for Industry* (Manchester: Association of Certifying Factory Surgeons, 1925), 8.

4 Richard Gillespie, ‘Industrial Fatigue and the Discipline of Physiology’, in John C. Wood and Michael C. Wood, eds, *George Elton Mayo: Critical Evaluations in Business Management* (London: Routledge, 2004), 429–57. See also, Steve Sturdy and Roger Cooter, ‘Science, Scientific Management, and the Transformation of Medicine in Britain c.1870–1950’, *History of Science*, 1998, 36, 448–9.

5 Anson Rabinbach, *The Human Motor: Energy, Fatigue, and the Origins of Modernity* (New York: Basic Books, 1990), 179–237.

6 Rabinbach, ‘The Body without Fatigue’.

7 Angelo Mosso, *Fatigue*, trans. Margaret Drummond and W. B. Drummond (London: Swan Sonnenschein & Co., 1904).

8 G. R. Searle, *The Quest for National Efficiency: A Study in British Politics and Political Thought, 1899–1914* (London: Ashfield Press, 1990), 1.

9 François Guéry and Didier Deleule, *The Productive Body* (Winchester: Zero Books, 2014).

10 See Karl Marx, *Capital: A Critique of Political Economy*, vol. 1 (London: Lawrence & Wishart, 1954), especially 222–86, 296–475.

11 Philip Barnard and Stephen Shapiro, ‘Editors’ Introduction to the English Edition’, in François Guéry and Didier Deleule, eds, *The Productive Body* (Winchester: Zero Books, 2014), 11.

12
*Ibid*., 12.

13 See Marx, *Capital*, 323–31.

14 See *ibid*., 477–85.

15 Guéry and Deleule, *The Productive Body*, 52.

16
*Ibid*., 106.

17 See *ibid*., 123.

18 See for example Steven Kreis, ‘Early Experiments in British Scientific Management: The Health of Munitions Workers Committee, 1915–1920’, *Journal of Management History*, 1995, 1, 65–78.

19 Bruno Latour, ‘On the Partial Existence of Existing and Nonexisting Objects’, in Lorraine Daston, ed., *Biographies of Scientific Objects: The Coming into Being and Passing Away of Scientific Objects* (Chicago: University of Chicago Press, 2000); Bruno Latour and Steve Woolgar, *Laboratory Life: The Construction of Scientific Facts* (Princeton, NJ: Princeton University Press, 1986), 105–7; Lorraine Daston, ‘The Coming into Being of Scientific Objects’, in Daston, ed., *Biographies of Scientific Objects*, 1–14; Ian Hacking, ‘Historical Ontology’, in *Historical Ontology* (Cambridge, MA: Harvard University Press, 2004), 1–26.

20 A. J. McIvor, ‘Employers, the Government, and Industrial Fatigue in Britain, 1890–1918’, *British Journal of Industrial Medicine*, 1987, 44, 724.

21 See McIvor, ‘Employers, the Government, and Industrial Fatigue’, 726–28; Steven Kreis, ‘The Diffusion of an Idea: A History of Scientific Management in Britain 1890–1945’ (PhD, University of Missouri-Columbia, 1990), 121–2.

22 William Mather, *The Forty-Eight Hours Week: A Year’s Experiment and Its Results at the Salford Iron Works, Manchester* (Manchester: Guardian Printing Works, 1894), 15.

23 Edward Cadbury, *Experiments in Industrial Organization* (New York: Longmans, Green, and Co., 1912), 246.

24 Searle, *The Quest for National Efficiency*, 60–7; Greta Jones, *Social Hygiene in Twentieth Century Britain* (London: Croom Helm, 1986), 15, 25.

25 Henry Jephson, *The Sanitary Evolution of London* (London: T. F. Unwin, 1907), 2.

26 Margaret Drummond, ‘Fatigue’, *The Strand Magazine*, 1905, 29, 217–21. Drummond was the translator, with her husband William, of the first English edition of Angelo Mosso’s *Fatigue*, published in 1904.

27
*XIIIe International Congrès D’Hygiène et de Démographie: Compte Rendu Du Congrès: Tome V: Première Division.—Hygiène. Section IV. Hygiène Industrielle et Professionnelle* (Bruxelles: P. Weissenbruch, Imprimeur Du Roi, 1903), 61.

28
*Annual Report of the Chief Inspector of Factories and Workshops for the Year 1903* (London: HMSO, 1904), Cd. 2193, 297. See also, ‘The Physiology of Fatigue', *British Medical Journal*, 1904, 1, 146.

29
*Ibid*., 296–7.

30
*Report of the Inter-Departmental Committee on Physical Deterioration*, Vol. I. (London: HMSO, 1904), 28, 87.

31 Josephine Goldmark, *Fatigue and Efficiency: A Study in Industry* (New York: Charities Publication Committee, 1912), 1; Gary S. Cross, *A Quest for Time: The Reduction of Work in Britain and France, 1840–1940* (Berkeley: University of California Press, 1989), 112–13.

32 B. L. Hutchins, ‘Fatigue and Efficiency’, *The Sociological Review*, 1913, 6, 30.

33
*Ibid*., 41–2; L. V. Lester-Garland, ‘Fatigue and Efficiency’, *The Economic Review*, 1913, 23, 226–9.

34
*Annual Report of the Chief Inspector of Factories and Workshops for the Year 1912* (London: HMSO, 1913), viii, xii; A. F. Stanley Kent, *Interim Report on an Investigation of Industrial Fatigue by Physiological Methods* (London: HMSO, 1915), 3.

35 Kent, *Interim Report*, 4.

36 A. F. Stanley Kent, *Second Interim Report on an Investigation of Industrial Fatigue by Physiological Methods* (London: HMSO, 1916), Cd. 8335, 50–60.

37 ‘Mental and Muscular Fatigue. Interim Report of the Committee’, in *Report of the Eightieth Meeting of the British Association for the Advancement of Science: Sheffield: 1910: August 31—September 7* (London: John Murray, 1911), 292–7; ‘Mental and Muscular Fatigue. Report of the Committee’, in *Report of the Eightieth [Eighty-First] Meeting of the British Association for the Advancement of Science: Portsmouth: 1911: August 31—September 7* (London: John Murray, 1912), 174–6.

38 Bodleian Library, BAAS 338, ‘Section F—Appointments to Committees 1888–1916’.

39 ‘The Question of Fatigue from the Economic Standpoint. Interim Report of the Committee’, in *Report of the Eighty-Fourth Meeting of the British Association for the Advancement of Science: Australia: 1914: July 28—August 31* (London: John Murray, 1915), 175–6.

40 Kent, *Second Interim Report*, 6; ‘The Question of Fatigue from the Economic Standpoint. Report of the Committee’, in *Report of the Eighty-Fifth Meeting of the British Association for the Advancement of Science: Manchester: 1915: September 7–11* (London: John Murray, 1916), 299.

41 McIvor, ‘Employers, the Government, and Industrial Fatigue’, 728–30.

42 David French, ‘The Military Background to the “Shell Crisis” of May 1915’, *Journal of Strategic Studies*, 1979, 2, 192–205; Peter Fraser, ‘The British “Shells Scandal” of 1915’, *Canadian Journal of History*, 1983, 18, 77–86.

43 R. J. Q. Adams, *Arms and the Wizard: Lloyd George and the Ministry of Munitions, 1915–1916* (London: Cassell, 1978).

44 HMWC, *Final Report: Industrial Health and Efficiency* (HMSO, 1918), Cd. 9056, 3.

45 See McIvor, ‘Employers, the Government, and Industrial Fatigue’, 730–1.

46 ‘War Workers’ Health’, *Daily Telegraph* (17 September 1915).

47 Thomas Bedford, ‘H. M. Vernon, M.A., M.D’, *British Journal of Industrial Medicine*, 1951, 8/2, 96–7. Vernon was not the only industrial physiologist with first-hand experience of factory work. Charles Sherrington, another Oxford physiologist, chair of the British Association Committee on Mental and Muscular Fatigue, and the first Chairman of the Industrial Fatigue Research Board in 1918, also volunteered in the Vickers-Maxim factory in Birmingham during the summer of 1915. See, H. M. Sinclair, ‘Sherrington and Industrial Fatigue’, *Notes and Records of the Royal Society*, 1984, 39/1, 91–104.

48 McIvor, ‘Employers, the Government, and Industrial Fatigue’, 730; Gillespie, ‘Industrial Fatigue and the Discipline of Physiology’, 439–40.

49 Henry John Spooner, *Industrial Fatigue in Its Relation to Maximum Output* (London: Co-partnership Publishers, 1917), 13.

50
*Ibid*., 16.

51 National Archives, MUN 5/92/346/7 (Memorandum: Health and Welfare of Munition Workers, 5 June 1917). The comparison of munition workers and soldiers was common. See, for example, HMWC, *[Memorandum No. 1:] Sunday Labour* (London: HMSO, 1915), Cd. 8132, 3; HMWC, *Interim Report: Industrial Efficiency and Fatigue* (HMSO, 1917), Cd. 8511, 77.

52 HMWC, *Health of the Munition Worker* (London: HMSO, 1917), 13.

53 National Archives, FD 3/120 (Newman to Fletcher, 24 May 1918).

54 See HMWC, *Memorandum No. 2: Welfare Supervision* (London: HMSO, 1915), Cd. 8151.

55 National Archives, FD 5/37 (Addison to Fletcher, 2 Sep. 1915). See also Gail Braybon, *Women Workers in the First World War: The British Experience* (London: Croom Helm, 1981), 138–2; Angela Woollacott, *On Her Their Lives Depend: Munitions Workers in the Great War* (Berkeley: University of California Press, 1994), 71–2.

56 HMWC, *Memorandum No. 7: Industrial Fatigue and Its Causes* (London: HMSO, 1916), Cd. 8213, 3.

57
*Ibid*.

58 H. M. Vernon, *Industrial Fatigue and Efficiency* (London: Routledge, 1921), 2; H. M. Vernon, ‘The Influence of Fatigue on Health and Longevity’, *Journal of Industrial Hygiene*, 1921, 3, 93–8.

59 Derickson, ‘Physiological Science and Scientific Management in the Progressive Era’, 487.

60 HMWC, *Memorandum No. 13: Juvenile Employment* (London: HMSO, 1916), Cd. 8362, 3.

61 HMWC, *Memo No. 7*, 6.

62 Interim Report, 4; Philip Sargant Florence, *Use of Factory Statistics in the Investigation of Industrial Fatigue* (New York: Columbia University, 1918).

63 HMWC, *Memo No. 7*, 6.

64 HMWC, *Memo No. 13*, 11.

65 See, Frederick Winslow Taylor, *The Principles of Scientific Management* (New York: Harper, 1913); Daniel Nelson, *Frederick W. Taylor and the Rise of Scientific Management* (Madison: University of Wisconsin Press, 1980); Robert Kanigel, *The One Best Way: Frederick Winslow Taylor and the Enigma of Efficiency* (Cambridge, MA: MIT Press, 2005).

66 ‘The Question of Fatigue from the Economic Standpoint. Interim Report’, 1916, 322. Emphasis added.

67 HMWC, *Final Report*, 42. Emphasis added.

68 A. F. Stanley Kent, ‘An Address on Fatigue and Alcohol’, *The Lancet*, 1917, 190, 107.

69 Michael Rose, *Industrial Behaviour: Research and Control* (London: Penguin, 1988), 61–6.

70 National Archives, MUN 5/92/346/7 (Memorandum: Health and Welfare of Munition Workers, 5 June 1917).

71 National Archives, FD 5/37 (Memorandum on Industrial Fatigue in Relation to Output, 14 July 1915). Emphasis added. In a letter of 1916, HMWC member Edgar Collis wrote to Fletcher, ‘your term “Physiological Management” hits me as fine; it covers our field as opposed to “Scientific Management” beautifully.’ *Ibid*. (Collis to Fletcher, 30 May 1916).

72 HMWC, *Memo No. 7*.

73
*Ibid*., 7.

74 A. F. Stanley Kent, ‘Industrial Fatigue’, in A. E. Berriman *et al*., *Industrial Administration: A Series of Lectures* (Manchester: Manchester University Press, 1920), 194.

75 J. R. Clynes, ‘Foreword’, in Henry John Spooner, *Industrial Fatigue in Its Relation to Maximum Output* (London: Co-partnership Publishers, 1917), 5–6.

76 David Harvey, *Spaces of Hope* (Edinburgh: Edinburgh University Press, 2000), 106. See also Jacques Donzelot, ‘Pleasure in Work’, in Graham Burchell, Colin Gordon, and Peter Miller, eds, *The Foucault Effect: Studies in Governmentality* (Chicago: University of Chicago Press, 1991), 260.

77 Steve Sturdy, ‘The Industrial Body’, in Roger Cooter and John Pickstone, eds, *Companion to Medicine in the Twentieth Century* (London: Routledge, 2003), 224–5; John Pickstone, ‘Production, Community and Consumption: The Political Economy of Twentieth Century Medicine’, in Roger Cooter and John Pickstone, eds, *Companion to Medicine in the Twentieth Century* (London: Routledge, 2003), 2–3.

78 Vicky Long, *The Rise and Fall of the Healthy Factory: The Politics of Industrial Health in Britain, 1914–60* (New York: Palgrave Macmillan, 2011), 2.

79
*Ibid*., 86.

80 Florence, *Use of Factory Statistics in the Investigation of Industrial Fatigue*, 19.

81 HMWC, *Health of the Munition Worker*, 13.

82 Excluding its appearance in the Committee’s terms of reference—reprinted in each report—the word ‘fatigue’ appears over 150 times across the twenty short memoranda nominally devoted to other subjects, and more than 400 times across the two larger reports.

83 HMWC, *Memorandum No. 8: Special Industrial Diseases* (London: HMSO, 1916), 3; HMWC, *Memorandum No. 10: Sickness and Injury* (London: HMSO, 1916), 3; Antonia Ineson and Deborah Thom, ‘T.N.T. Poisoning and the Employment of Women Workers in the First World War’, in Paul Weindling, ed., *The Social History of Occupational Health* (London: Croom Helm, 1985), 89–107.

84 Arthur McIvor, ‘Manual Work, Technology, and Industrial Health, 1918–39’, *Medical History*, 1987, 31, 162; Long, *The Rise and Fall of the Healthy Factory*.

85
*Interim Report*, 69, 70.

86 HMWC, *Memo No. 7*, 3.

87
*Ibid*., 4–5; Florence, *Use of Factory Statistics in the Investigation of Industrial Fatigue*, 23.

88 Harry E. Mock, ‘Industrial Medicine and Surgery—A Résumé of Its Development and Scope’, *Journal of Industrial Hygiene*, 1919, 1, 1.

89 HMWC, *Final Report*, 7.

90
*Annual Report of the Chief Inspector of Factories and Workshops for the Year 1918* (London: HMSO, 1919), Cmd. 340, iv; National Archives, LAB/2419/HQ/844/1918 (Memorandum: Industrial Fatigue Research Board); ‘Industrial Fatigue’, *The Times*, 20 December 1918.

91 Gail Braybon, *Women Workers in the First World War*, 116; McIvor, ‘Employers, the Government, and Industrial Fatigue’.

## Notes

Steffan Blayney is a Wellcome Trust-funded doctoral candidate in the department of History, Classics & Archaeology at Birkbeck, University of London. His research looks at fatigue, the science of work, and the working body in Britain, *c*. 1870–1939.

